# Familial and genetic overlap between Sjögren’s disease and other autoimmune diseases

**DOI:** 10.3389/fimmu.2026.1740360

**Published:** 2026-03-26

**Authors:** Hitomi Ono-Minagi, Takuma Ohnishi, Natalie Atyeo, Kentaro Okuno, Peter D. Burbelo, John A. Chiorini

**Affiliations:** 1 Adeno-Associated Virus Biology Section, National Institute of Dental and Craniofacial Research, National Institutes of Health, Bethesda, MD, United States; 2 Advanced Research Center for Oral and Craniofacial Sciences Dental School, Okayama University Graduate School of Medicine, Dentistry and Pharmaceutical Sciences, Okayama, Japan; 3 Department of Pediatrics, Keio University School of Medicine, Tokyo, Japan; 4 Department of Geriatric Dentistry, Osaka Dental University, Osaka, Japan

**Keywords:** autoimmune disease, familial risk, gene variant, genetic predisposition, risk factors, Sjögren’s syndrome

## Abstract

**Purpose:**

To evaluate familial clustering of Sjögren’s disease (SjD) with other autoimmune diseases and to characterize shared genetic architecture using an integrative genomic approach.

**Methods:**

Meta-analysis identified studies assessing autoimmune disease incidence in probands and first-degree relatives (FDRs), and pooled relative risks (RRs) were calculated. Publicly available genome-wide association study (GWAS) summary statistics were analyzed within a hierarchical genetic architecture framework integrating genome-wide polygenic correlation (LDSC), locus-level overlap (Jaccard index), union-based susceptibility mapping, and SNP-level pleiotropy detection.

**Results:**

Eighteen studies evaluated familial aggregation of SjD and other autoimmune diseases, of which nine were included in the pooled analysis. The RR of SjD was 10.54 when both proband and FDR were affected. Among discordant autoimmune probands, systemic lupus erythematosus (SLE) showed the highest RR for SjD (4.49), followed by systemic sclerosis (2.65). Genome-wide analyses demonstrated substantial polygenic sharing between SjD and systemic autoimmune diseases, positioning SjD as a bridging disorder. However, locus-level overlap was largely driven by the HLA region; after HLA exclusion, shared loci markedly decreased and were restricted to a limited number of immune regulatory hubs. SNP-level pleiotropy analyses similarly indicated predominantly HLA-dependent sharing with fewer non-HLA signals.

**Conclusion:**

SjD shows strong familial aggregation and shared genetic susceptibility with multiple autoimmune diseases. These findings support a hierarchical model in which broad HLA-driven polygenic sharing coexists with selective non-HLA convergence.

**Strengths and limitations of this study:**

This study integrates systematic familial meta-analysis with GWAS data to characterize shared autoimmune genetic architecture. However, inference regarding shared causal variants remains limited by reliance on summary statistics and locus-based resolution.

## 1 Introduction

Sjögren’s disease (SjD; encompassing primary Sjögren’s syndrome) is a complex systemic autoimmune disease affecting millions worldwide (1). While classically characterized by exocrine dysfunction causing dry mouth and dry eyes, SjD is increasingly recognized as a multisystem disorder extending beyond the exocrine glands (2, 3). Patients with SjD have an increased risk of B-cell lymphoma, most commonly indolent extranodal marginal zone/MALT lymphoma, with prognosis varying according to histologic subtype and clinical risk factors. In addition, fatigue and extraglandular involvement contribute substantially to disease burden and reduced quality of life (4–6). SjD likely arises from the multifactorial interplay of environmental, hormonal, infectious, and genetic factors, among which genetics plays a pivotal role (3, 7–9). Notably, GWAS comparing SjD patients with healthy controls have identified several disease-associated loci (10, 11). Notably, the Human Leukocyte Antigens (HLA) class II region, particularly HLA-DRB1, is one such gene region that has been strongly implicated in SjD susceptibility. Polymorphisms in many other genes involved in immune function, including Interferon Regulatory Factor 5 (IRF5), Signal Transducer and Activator of Transcription 4 (STAT4), and Tumor Necrosis Factor Alpha-Induced Protein 3 (TNFAIP3) have also been linked to SjD (12–14). However, many of these loci are influenced by ethnicity and no single marker is indicative of disease. Furthermore, many rare genetic risk variants are found in non-coding regions, making their functional role difficult to determine (15). Large population-based data further support substantial non-random co-occurrence of autoimmune diseases at the individual level across the life course (16).

SjD is commonly categorized as primary (pSjD), when SjD occurs as the predominant autoimmune condition, and secondary (sSjD), when SjD features occur in the context of another systemic autoimmune disease such as rheumatoid arthritis (RA), systemic lupus erythematosus (SLE), or primary biliary cholangitis (PBC). Importantly, pSjD is not limited to exocrine glands in which extraglandular manifestations including fatigue, arthralgia, and pulmonary, renal, neurologic, or cutaneous involvement are well recognized and contribute substantially to morbidity. Because universally accepted classification criteria for sSjD are not established across settings, sSjD is variably defined in research; in clinical practice, sicca features in the context of an established systemic autoimmune disease are typically identified by clinical assessment of sicca features and objective glandular findings within a patient who already carries another systemic autoimmune diagnosis.

Several lines of evidence suggest an association between SjD and autoimmune disease within families. Case reports have described shared SjD diagnoses in first degree relatives (FDRs) of patients with the condition (17). These reports mostly revealed shared SjD diagnoses between sibling groups (6) and described co-occurring SjD-related conditions in families, including parotid lymphoma and hemolytic anemia. A 1984 study by Revenelle et al. examined a small cohort of individuals with pSjD or sSjD in Maryland (18). Among the 51 individuals with pSjD, seven had a family history of SjD. Further, 30–35% of individuals with SjD in the cohort also had a family history of other autoimmune conditions, including SLE, RA and thyroid disease. Other studies have focused on small groups of autoimmune cases in specific geographical regions, making it difficult to draw generalizable conclusions (19). While it is generally assumed that a positive family history of SjD predisposes individuals to other autoimmune diseases, the actual risk across diverse populations and diseases is unknown. A meta-analysis by Jin et al. suggested that familial aggregation of autoimmune diseases may contribute to the risk of developing SjD, although mechanistic details were not addressed (20). Several large national registry studies have provided valuable estimates of familial risk within individual autoimmune diseases (14). These studies have typically focused on single-disease aggregation and did not directly compare cross-disease familial risks. Therefore, it remains unclear whether the genetic predisposition underlying SjD overlaps quantitatively with those of other autoimmune conditions. To date, no comprehensive meta-analysis has been conducted to integrate familial risk of SjD across different autoimmune diseases to assess a potential genetic predisposition (18, 21, 22).

Here, we integrate data from multiple national registry studies and harmonize relative risk (RR) estimates across diseases to uniquely quantify cross-disease familial aggregation and establish a comparative hierarchy of autoimmune relationships. The objectives were to evaluate the association between SjD and family history of autoimmune disease in FDRs of individuals with SjD and individuals with FDRs affected by SjD.

## 2 Methods

### 2.1 Search strategy and eligibility criteria for meta-analysis

An electronic literature search in PubMed was performed on 16 February 2025 using pre-specified search terms (Appendix 1) to identify studies related to SjD, autoimmune disease and familial risk covering the period from 1 January 1980 to 16 February 2025. Covidence (Veritas Health Innovation, Melbourne, Australia) was used to extract and review articles and identify duplicate entries (www.covidence.org). Manual searches of the reference lists of search term-identified review articles were conducted to confirm that our search terms comprehensively identified all topical articles.

Two authors (H.OM., and T.O.) independently screened all extracted abstracts to determine the eligibility of each article for further analysis. Conflicts were resolved through a third assessor (N.A.). Abstracts were excluded if they (a) were not topical (did not focus on autoimmune disease, genetics, inheritance or familial relationships) (b) were exclusively animal studies (c) focused on a specific genetic locus (d) were not available in full-text format or (e) were not published in the English language.

After this initial abstract review, two authors (H.OM., and T.O.) independently evaluated the full texts of selected abstracts. To create a narrower focus for downstream analysis, the list of autoimmune disease targets was restricted to nine well-characterized autoimmune disorders with defined disease-specific autoantibodies and established research classification criteria and/or validated case definition included: SjD, SLE, systemic sclerosis (SSc), Rheumatoid Arthritis (RA), idiopathic inflammatory myopathies (IIM), multiple sclerosis (MS), type I diabetes (T1D), Graves’ Disease (GD) and Hashimoto’s Thyroiditis (HT). Exclusion criteria included the lack of a SjD group, lack of a control (no autoimmune disease) group, no description of FDR relationships, and incomplete reporting of standard incidence ratio and RR without sufficient data points to calculate these values. Although several autoimmune diseases were included in the epidemiological meta-analysis, only diseases with available genome-wide summary statistics suitable for cross-trait genetic architecture analyses were included in the GWAS-based analyses.

### 2.2 Bias assessment

The methodological qualities of all included studies were assessed and scored with the Risk of Bias Assessment tool for Non-Randomized Studies 2 (RoBANS2) (23, 24). This tool is designed to rate the risk of bias for nonrandomized studies, and comprises eight criteria: selection of participants, target group selection, confounding variables, measurement of exposure, blinding of outcome assessments, outcome assessment, incomplete outcome data, and selective outcome data. Each domain is judged to be ‘Low,’ High,’ or ‘Unclear’. Two reviewers (H. OM. and T. O.) independently assessed the risk of bias, and any discrepancies were resolved through discussion. Inter-rater reliability was evaluated using Cohen’s κ coefficient. Agreement between reviewers was moderate during the title and abstract screening (κ = 0.43) and improved during the full-text review (κ = 0.52), indicating moderate-to-substantial consistency. To assess the risk of publication bias in the articles that were further selected for meta-analysis, Egger’s and Begg’s test were constructed using the standard error and the difference of mean values (**Supplementary Table 1**).

### 2.3 Methodological approach for meta-analysis

Analyses of the selected articles were conducted by three authors (H.OM., T.O., and N.A.). First, each unique disease association described in the article was treated as an individual study. For example, if an article described associations between SjD in the proband and SLE and GD in the FDRs, both the SjD->SLE relationship and SjD->GD relationships were analyzed separately. Similarly, if the article distinguished parent and sibling FDRs, each association (proband->sibling and proband->parent) was analyzed separately. In this study, “proband” refers to the index case, that is, the individual diagnosed with SjD or another autoimmune disease serving as the starting point of familial aggregation analysis. Familial associations were categorized as “concordant” when both the proband and their first-degree relative (FDR) were affected by the same autoimmune disease (e.g., proband = SjD, FDR = SjD), and as “discordant” when they were affected by different autoimmune diseases (e.g., proband = SLE, FDR = SjD). Importantly, only associations in which either the FDR group or proband group were diagnosed with SjD were considered. Next, the number of cases and controls were extracted to calculate RR ratios. For clarity, “case” refers to a member of the proband group diagnosed with the autoimmune disease of interest, and “control” refers to a member of the proband group that does not have the autoimmune disease of interest. Because the definition of the control group differed between the national cohort (population-based) and the individual case-control studies, we considered the individuals in case-control studies who did not meet the case definition as controls, regardless of their overall health status. Finally, RR of having FDRs with concordant or discordant autoimmune disease was calculated using the following formula:

RR= (a/(a+c))/(b/(b+d)).

where:

a = number of cases with FDRs with autoimmune disease.
b = number of controls with FDRs with autoimmune disease.
c = number of cases without FDRs with autoimmune disease.
d = number of controls without FDRs with autoimmune disease.

### 2.4 Synthesized meta-analysis

To analyze relationships between proband autoimmune disease status and FDR disease incidence, select studies were included for meta-analysis. Among the 18 articles that met the initial inclusion criteria, 10 articles included sufficient data for quantitative meta-analysis. Five articles (Hemminki et al., (25); Hemminki et al., (26); Kuo et al., (27); Kuo et al., (28); Thomsen et al., (29)) were excluded because they analyzed the same diseases in overlapping cohorts. The analyses of the SjD probands and the RA FDR and GD FDR were based on Swedish national data in both studies (Hemminki et al., (25); Hemminki et al., (26); Thomsen et al., (29) Thomsen et al., (29)), so meta-integration was not possible. In cases where national cohorts from Sweden or Taiwan overlapped, we included only the article with the larger number of probands. Two articles (Alexandra et al., (30); Mescheriakova et al., 31) were excluded because they assessed the prevalence of the disease based on familial aggregation, meaning they counted cases within families already affected by autoimmune diseases. This approach could lead to an overestimation of prevalence and does not reflect population-based sampling. One article was excluded because it was the only study analyzing the specific disease association (32), preventing a pooled analysis. Last, one article (Priori et al., (33)) included zero proband cases and thus could not be incorporated into the meta-analysis (33).

For the 10 included studies, the RRs and their standard errors were used to calculate the weighted mean difference. The amount of difference reported in each article was evaluated from the weighted mean difference and its 95% confidence intervals (CI). A forest plot was constructed using the weighted mean difference of the target variable obtained by comparing a proband with familial autoimmune disease and a proband with non-familial (sporadic) autoimmune disease as a control. Meta-analysis was performed with the random and common effects model because the measurement methods, participants, and duration of the studies were different. Heterogeneity was assessed with the *I^2^* index and the tau-squared test. The *I^2^* represents the percentage of variability in a set of effect sizes that is due to heterogeneity rather than by chance. A value of *I^2^* less than 50% indicates low-to-moderate heterogeneity in which a fixed-effects model can be applied. This approach allowed for systematic assessment of the autoimmune association between proband and families with autoimmune diseases while accommodating the methodological variability of the studies included. If heterogeneity was identified, *post-hoc* subgroup analyses were performed based on the duration in the studies. Data analysis was performed using R Studio (https://posit.co/) with R version 4.2.2.

### 2.5 Selection of GWAS datasets and inclusion criteria

To investigate shared genetic architecture between SjD and other autoimmune diseases, we performed comparative analyses using publicly available GWAS summary statistics. Autoimmune diseases were selected based on prior literature reporting epidemiological or genetic associations with SjD, as well as their classification as representative autoimmune diseases characterized by well-established, disease-specific autoantibodies, and the availability and quality of GWAS data. GWAS datasets were included according to the following criteria: (i) the disease phenotype was independently and clinically defined (i.e., not an aggregate ICD-based phenotype); (ii) the study employed a case–control GWAS design (excluding burden tests, polygenic risk score analyses, or phenotype-wide association studies); (iii) full GWAS summary statistics were publicly available; and (iv) the GWAS was based on multiple cohorts or large-scale meta-analyses. To minimize confounding due to population stratification, we restricted analyses to GWAS datasets derived from European ancestry populations. In addition, preference was given to the most recent GWAS releases and studies with the largest available case numbers in order to maximize statistical power and ensure improved imputation quality and updated phenotype definitions. Where available, datasets from the same large-scale resource (FinnGen Release 12: https://www.finngen.fi/en/access_results) were selected to ensure consistency in phenotype definition and analytical pipelines. Based on these criteria, eight autoimmune diseases were included. In addition, diseases were selected based on prior literature classifying them as prototypical autoimmune disorders characterized by disease-specific autoantibodies (34). All datasets were lifted over to GRCh38 prior to locus definition.

The following GWAS datasets were selected from the GWAS Catalog (https://www.ebi.ac.uk/gwas/).

SjD: M13_SJOGREN, FinnGen (35).
GD: E4_GRAVES_STRICT, FinnGen (35).
RA: M13_RHEUMA, FinnGen (35).
MS: G6_MS, FinnGen (35).
HT: E4_THYROIDITAUTOIM, FinnGen (35).
T1D: Sakaue S. et al., GCST90014023 (36).
SLE: Bentham J. et al., GCST003156 (37).
PBC: Cordell H.J. et al., GCST90061440 (38).

### 2.6 Definition of genome-wide significant loci

For each disease, genome-wide significant (GWS) variants were defined as single nucleotide polymorphisms with a P value < 5 × 10^−8^. Because multiple GWS variants may arise from a single underlying association signal due to linkage disequilibrium (LD), analyses were conducted at the locus level rather than at the variant level (39, 40). To define independent GWS loci, a ±250 kb window was constructed around each GWS variant. Within each disease, overlapping windows were merged into a single continuous genomic interval. The resulting non-overlapping intervals were defined as independent GWS loci. Given the well-established and disproportionately strong contribution of the HLA region to autoimmune disease susceptibility, loci overlapping the HLA region were analyzed separately. The HLA region was defined as chromosome 6: 25–34 Mb (GRCh38) (41). Loci overlapping this region were classified as HLA loci, whereas all remaining loci were classified as non-HLA loci and retained for non-HLA–specific analyses.

### 2.7 Locus-based overlap and Jaccard similarity analysis

This analysis was designed to quantify overlap at the level of discrete GWS loci, complementing genome-wide polygenic correlation analyses. To quantify genetic overlap between autoimmune diseases, locus-based overlap analyses were performed using the sets of independent GWS loci. In addition to pairwise overlap metrics, a union-based framework was implemented in which all disease-specific loci were pooled and merged to generate a unified set of non-overlapping genomic regions (union loci) across all diseases. Pairwise locus overlap was quantified by counting shared union loci as well as by calculating the Jaccard similarity index, defined as the number of shared loci divided by the total number of loci across each disease pair. Jaccard indices were calculated using (i) all loci and (ii) non-HLA loci only, allowing assessment of the contribution of the HLA region to cross-disease genetic sharing. Differences between all-locus and non-HLA Jaccard indices were used to quantify the extent to which locus overlap was driven by the HLA region.

### 2.8 Assessment of disease-specific locus architecture

GWS variants (P < 5 × 10^−8^) were expanded by ±250 kb and merged to define independent loci, such that adjacent association signals in strong linkage disequilibrium were consolidated into a single locus. Accordingly, a locus represents an independent association peak rather than an individual variant. To systematically evaluate shared risk architecture across the eight autoimmune diseases, all disease-specific loci were pooled and merged if their genomic intervals overlapped, resulting in a unified set of non-overlapping genomic regions. Each union locus was counted once regardless of the number of overlapping disease-specific signals within that region. For each union locus, the presence or absence of genome-wide significant association for each disease was encoded as a binary variable (1 = at least one GWS SNP within the locus; 0 = no GWS SNP), generating a locus-by-disease binary matrix. Overlap among independent GWS loci across diseases was assessed using a union-based framework and visualized using Venn diagrams constructed from the full set of loci (including HLA loci). Union loci were constructed by merging loci across all diseases, and each union locus was counted once regardless of the number of overlapping disease-specific signals within that region. To illustrate representative union loci in SjD, three genomic regions were highlighted: a non-HLA locus on chromosome 2 encompassing the STAT4 region, the HLA locus on chromosome 6, and a non-HLA locus on chromosome 7. Regional association plots were generated for each locus, displaying −log_10_(P) values across a ±250 kb window for all included diseases, with gene annotations shown below each plot.

### 2.9 SNP-level pleiotropy analysis using PLACO

To identify genetic variants exhibiting pleiotropic effects between autoimmune diseases, we applied PLACO (42). PLACO is designed to detect variants that are simultaneously associated with two traits by testing against a composite null hypothesis that includes scenarios where a variant affects neither trait or only one trait. Under this framework, the composite null hypothesis (H_0_) consists of three possibilities: (i) the variant is associated with neither trait, (ii) the variant is associated with trait 1 only, or (iii) the variant is associated with trait 2 only. The alternative hypothesis corresponds to true pleiotropy, in which the variant influences both traits. This approach reduces false-positive findings that arise when a strong association in one trait induces apparent but spurious association in another.

### 2.10 Implementation and extension (PLACO with cross-trait correlation adjustment)

To improve robustness under real GWAS conditions, we implemented a variance- and correlation-adjusted extension of PLACO (hereafter referred to as PLACO). This extension accounts for deviations from idealized assumptions of standard normal Z-scores, including variance inflation and cross-trait correlation due to shared controls or sample overlap. For each disease pair, allele-aligned Z-scores (Z_1_, Z_2_) were used to compute the PLACO test statistic (T_PLACO) and corresponding p-value (P_PLACO). Variance parameters (VarZ) for each trait and cross-trait correlation (CorZ) were estimated from approximately null SNPs using a screening threshold (p_screen = 1×10^−4^).

## 3 Results

### 3.1 Article selection

**Figure 1:**
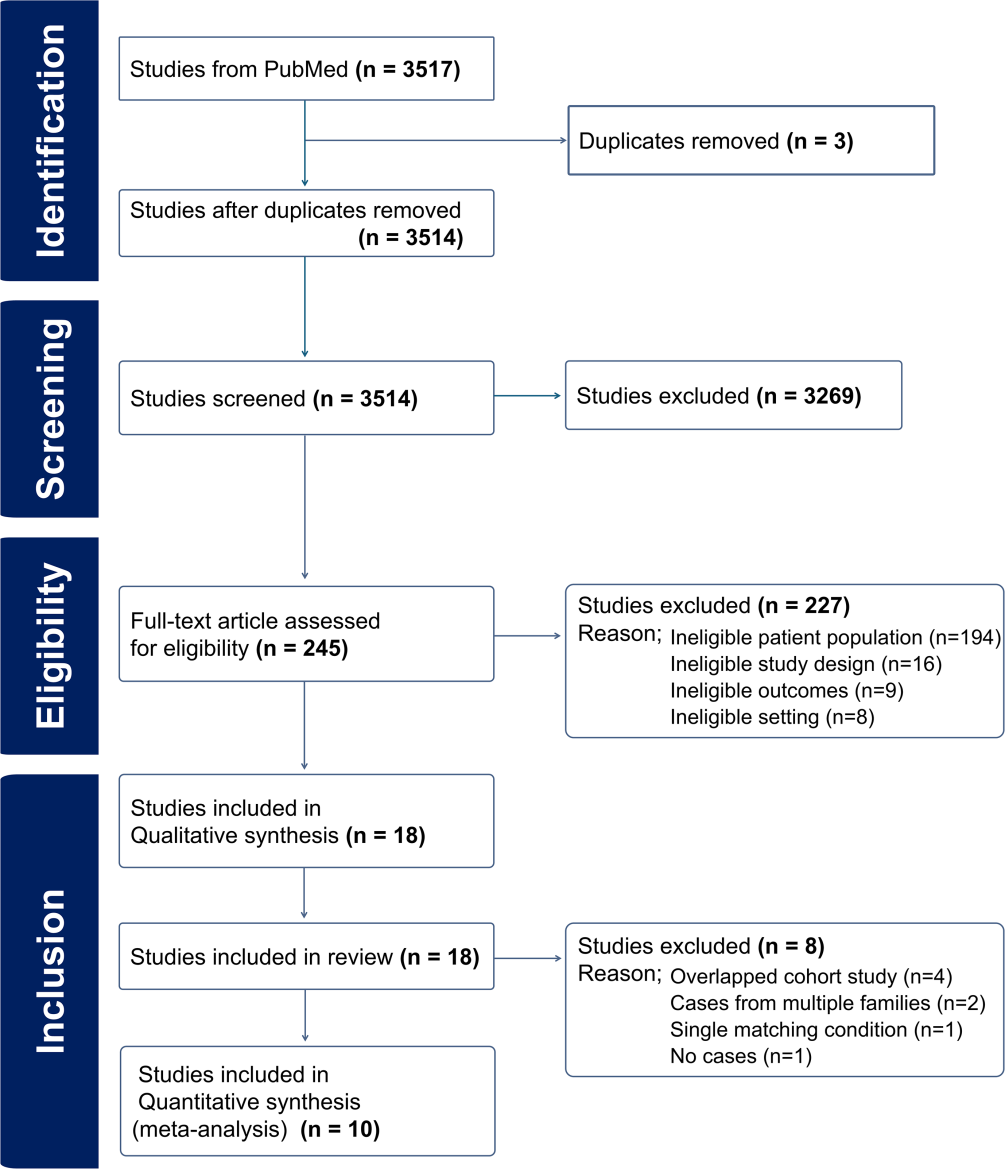
PRISMA diagram. Adapted Preferred Reporting Items for Systematic Reviews and Meta-Analyses (PRISMA) flow chart representing the study selection process.

The workflow for article selection is described in **Figure 1**. From a search of the PubMed database, 3517 relevant articles were retrieved. After removing duplicates, 3514 articles remained. After abstracts were assessed, 3269 publications were excluded, leaving 245 articles for full-text analysis. Of these 245 articles, 227 articles were excluded because the patient population did not align with our inclusion criteria. For example, articles were excluded if they did not have a proband group, focused on non-autoimmune conditions or did not analyze SjD in either the family or proband group. In addition, some were excluded due to reporting inconsistencies, such as failure to clearly distinguish between affected individuals and their relatives. The remaining 18 articles met the inclusion criteria for further analysis. Extracted studies were classified according to publication year, country of the study population, study design, autoimmune disease group of the proband, autoimmune disease groups of the FDRs, familial relationship of the FDRs, and method of SjD diagnosis.

Characteristics of the studies are described in **Table 1** and included analysis for multiple, other autoimmune diseases including SLE, SSC, RA, Idiopathic inflammatory myopathy (IIM), T1D, and MS. The articles included cohort studies and case control studies published between 1998–2020 and covered cohorts spanning four continents and provided sufficient data to analyze fifteen distinct SjD associations.

**Table 1:** Characteristics of studies included in systematic review and meta-analysis.

	Author	Year	Country	Study	Diseases		SjD diagnostic criteria
					Proband	FDRs	
1	Ginn (43)	1998	USA	Case Control Study	IIM	SjD	Confirmed by specialist, and records
2	Bengtsson (44)	2002	Swedish	Case Control Study	SLE	SjD	Confirmed by questionnaire
3	Priori (33)	2003	Italy	Case Control Study	SLE	SjD	Confirmed by specialist, treatment, and records
4	Anaya (45)	2006	Colombia	Case Control Study	SjD	AD	AECG
5	Eaton (46)	2007	Denmark	Registry-based Cohort Study	SjD	SjD	ICD
6	Nielsen (32)	2008	Denmark	Registry-based Cohort Study	MS	SjD	ICD
7	Hemminki (25)	2009	Sweden	Registry-based Cohort Study	SjD	RA	ICD
8	Hemminki (26)	2010	Sweden	Registry-based Cohort Study	SjD	GD	ICD
9	Priori (47)	2007	Italy	Case Control Study	SjD	AD	AECG
10	Kuo (27)	2015a	Taiwan	Registry-based Cohort Study	SjD, RA, SLE, IIM,	SjD, RA, SLE, SSc, IIM, T1D, MS	ICD
11	Kuo (48)	2015b	Taiwan	Registry-based Cohort Study	SLE	SjD	ICD
12	Kuo (49)	2016	Taiwan	Registry-based Cohort Study	SSc	SjD	ICD
13	Kuo (28)	2018	Taiwan	Registry-based Cohort Study	T1D	SjD	ICD
14	Alexandra (30)	2018	Israel	Registry-Based Case-Control Study	SSc*	SjD	AECG
15	Ben-Eli (50)	2019	Israel	Case Control Study	SjD	AD	AECG
16	Mescheriakova (31)	2019	Netherlands	Multigenerational Cohort Study	MS*	SjD	Confirmed by questionnaire
17	Thomsen (29)	2020a	Swedish	Registry-based Cohort Study	SjD, AD, RA, SLE, SSc, IIM, GD, HT	SjD, AD, RA, SLE, SSc, IIM, GD, HT	ICD
18	Thomsen (51)	2020b	Sweden	Registry-based Cohort Study	HT	SjD	ICD

Year, year of publication; FDRs, first-degree relative; *, multiplex families.

SjD, Sjögren’s disease; AD, autoimmune disease; SLE, systemic lupus erythematosus.

RA, rheumatoid arthritis; SSc, systemic sclerosis; IIM, idiopathic inflammatory myopathies.

T1D, type 1 diabetes; MS, multiple sclerosis; GD, Graves’ Disease; HT, Hashimoto’s Thyroiditis.

AECG, American-European Consensus Group; ICD, International classification of Diseases.

### 3.2 Bias assessment of selected studies

A total of eighteen studies were assessed and scored according to the RoBANS guidelines (34). **Table 2** presents the results of this assessment. Of these, three studies (Bengtsson et al., (44); Priori et al., (33); Alexandra et al., (30)) did not match cases and controls by age and sex, raising concerns about imbalance between groups; therefore, the risk of selection bias was rated as High in these studies (30, 44, 47). Regarding target group selection, two studies (Bengtsson et al., (44); Alexandra et al., (30)) diagnosed SjD based solely on physicians’ clinical judgment without standardized criteria, leading to a high risk of bias due to the potential unreliability of diagnosis (30, 44). In terms of confounding factors, only two studies (Mescheriakova et al., (31); Ben-Eli et al., (50)) accounted for or adjusted by stratification and thus were rated as Low risk (31, 50). For measurement of exposure, three older studies (Ginn et al., (43); Bengtsson et al., (44); Priori et al., (33)) relied solely on self-reported family history obtained through interviews without verification (33, 43, 44). These were judged as having a high risk due to potential misclassification. With respect to blinding of assessors, most case-control studies were rated as High risk, as family history was often recorded by assessors who were aware of the proband’s disease status (30, 31, 44, 45, 47, 50). Concerning incomplete outcome data, three studies (Bengtsson et al., (44); Mescheriakova et al., (31); Ben-Eli et al., (50)) did not provide sufficient information on how missing data were handled, leading to a high-risk assessment (30, 31, 44). Finally, for selective outcome reporting, all studies reported data regarding not only family history but also other data, including demographic or clinical characteristics, and therefore the risk was rated as Low.

**Table 2:** Quality assessment score (RoBANS).

Author	Year	Risk of bias							
		Selection bias	Target group	Confounding variables	Measurement of exposure	Blinding assessment	Outcome assessment	Incomplete outcome	Selective outcome
Ginn (43)	1998	Low	Low	Highc	Highd	Low	Low	Low	Low
Bengtsson (44)	2002	Higha	Highb	Highc	Highd	Highe	Highf	Highg	Low
Priori (33)	2003	Low	Low	Highc	Highd	Low	Low	Low	Low
Anaya (45)	2006	Low	Low	Highc	Low	Highe	Low	Low	Low
Priori (47)	2007	Higha	Low	Highc	Low	Highe	Low	Low	Low
Eaton (46)	2007	Low	Low	Highc	Low	Low	Low	Low	Low
Nielsen (32)	2008	Low	Low	Highc	Low	Low	Low	Low	Low
Hemminki (25)	2009	Low	Low	Highc	Low	Low	Low	Low	Low
Hemminki (26)	2010	Low	Low	Highc	Low	Low	Low	Low	Low
Kuo (27)	2015a	Low	Low	Highc	Low	Low	Low	Low	Low
Kuo (48)	2015b	Low	Low	Highc	Low	Low	Low	Low	Low
Kuo (49)	2016	Low	Low	Highc	Low	Low	Low	Low	Low
Mescheriakova (31)	2018	Low	Low	Low	Low	Highe	Highf	Highg	Low
Kuo (28)	2018	Low	Low	Highc	Low	Low	Low	Low	Low
Alexandra (30)	2019	Higha	Highb	Highc	Low	Highe	Highf	Highg	Low
Ben-Eli (50)	2019	Low	Low	Low	Low	Highe	Low	Low	Low
Thomsen (29)	2020a	Low	Low	Highc	Low	Low	Low	Low	Low
Thomsen (51)	2020b	Low	Low	Highc	Low	Low	Low	Low	Low

Year, year of publication; RoBANS, the Risk of Bias Assessment Tool for Nonrandomized Studies.

a Age and gender not matched.

b Diagnosis of Sjögren’s disease is unreliable.

c Confounders were not confirmed.

d Self-reported family history.

e Not blinded to assessors.

f Unsystematic family history ascertainment.

g Data is significantly under-reported or missing data is poorly handled.

h Only data showing significant differences from the study results is shown.

### 3.3 Relative risk of SjD and other autoimmune diseases within families

RRs for SjD or other autoimmune disease in FDRs of SjD probands are described in **Table 3**. Among all autoimmune diseases, concordant SjD was the most common autoimmune disease in FDRs of SjD probands (study-specific RRs; 8.64-12.77). Sub-analysis on familial aggregation of SjD in siblings versus parents showed that siblings (study-specific RRs; 9.36-16.59) demonstrated higher RRs compared to parents (study-specific RRs; 7.95-14.79), suggesting stronger familial aggregation of SjD between siblings.

**Table 3:** Studies of familial autoimmune diseases in SjD probands.

Disease		Author	Year	Country	Study	Relative	Case(n)	RR	logRR	SE (logRR)	95% CI
Probands	FDRs										
SjD	SjD	Eaton (46)	2007	Denmark	Registry-based Cohort Study	1st degree	16	12.77	2.55	0.31	7.00-23.30
						Parents	–	14.79	2.69	0.31	8.10-27.00
						Sibling	–	16.59	2.81	0.56	4.10-67.10
		Kuo (27)	2015a	Taiwan	Registry-based Cohort Study	1st degree	105	12.37	2.52	0.13	9.54-16.05
						Parents	47	12.46	2.52	0.15	9.34–16.62
						Sibling	40	18.99	2.94	0.33	9.76–36.93
		Thomsen (29)	2020a	Sweden	Registry-based Cohort Study	1st degree	177	8.64	2.16	0.077	7.44-10.05
						Parents	70	7.95	2.07	0.13	6.22-10.16
						Sibling	109	9.36	2.24	0.099	7.71-11.37
SjD	AD	Anaya (45)	2006	Colombia	Case Control Study	1st degree	38	1.73	0.55	0.3	0.96-3.11
		Priori (47)	2007	Italy	Case Control Study	1st degree	33	5.14	1.64	0.5	1.93-13.67
		Ben-Eli (50)	2019	Israel	Case Control Study	1st degree	33	4.03	1.39	0.32	2.13-7.61
		Thomsen (29)	2020a	Sweden	Registry-based Cohort Study	1st degree	158	8.58	2.15	0.08	7.34-10.03
SjD	RA	Hemminki (25)	2009	Sweden	Registry-based Cohort Study	Parents	11	2.12	0.75	0.33	1.11–4.03
						Sibling	8	2.25	0.81	0.57	0.74–6.84
		Thomsen (29)	2020a	Sweden	Registry-based Cohort Study	1st degree	409	1.77	0.57	0.049	1.61-1.95
SjD	SLE	Thomsen (29)	2020a	Sweden	Registry-based Cohort Study	1st degree	46	2.77	1.02	0.15	2.07-3.71
SjD	SSc	Thomsen (29)	2020a	Sweden	Registry-based Cohort Study	1st degree	15	2.06	0.72	0.26	1.23-3.46
SjD	IIM	Thomsen (29)	2020a	Sweden	Registry-based Cohort Study	1st degree	5	0.76	-0.27	0.48	0.30-1.94
SjD	GD	Hemminki (26)	2010	Sweden	Registry-based Cohort Study	Parents	5	1.06	0.061	0.51	0.39-2.90
						Sibling	2	0.60	-0.50	1.10	0.07-5.24
		Thomsen (51)	2020b	Sweden	Registry-based Cohort Study	1st degree	109	1.22	0.21	0.095	1.02-1.48

FDR, first-degree relative; Year, year of publication.

SjD, Sjögren’s disease; AD, autoimmune diseases; SLE, systemic lupus erythematosus; RA, rheumatoid arthritis.

SSc, systemic sclerosis; IIM, idiopathic inflammatory myopathies; GD, Graves’ Disease.

Case, SjD positive with AD FDR; RR, relative risk.

FDRs of SjD probands also had an increased risk of autoimmune disease in general, with RR ranging from 1.73 to 8.58. Specifically, FDRs had an increased risk of RA, SLE and SSc, and no conclusive elevated risk for GD or IIM. RRs of SjD in FDRs of probands with any autoimmune disease are described in **Table 4**. SLE was most highly associated with SjD in FDRs (study-specific RRs; 3.22-6.85), while probands with RA, SSc and MS all had moderate RR of SjD in FDRs. Individuals with T1D, GD and HT thyroiditis had little to no increased RR in FDRs with SjD, while probands with IIM showed a very low RR for having FDRs with SjD.

**Table 4:** Studies of probands of different autoimmune diseases with FDR with SjD.

Disease		Author	Year	Country	Study	Relative	Case	RR	logRR	SE (logRR)	95% CI
Probands	FDRs										
RA	SjD	Kuo (27)	2015a	Taiwan	Registry-based Cohort Study	1st degree	68	2.95	1.08	0.12	2.33-3.73
		Thomsen (29)	2020a	Sweden	Registry-based Cohort Study	1st degree	243	1.69	0.52	0.065	1.49-1.91
SLE	SjD	Bengtsson (44)	2002	Sweden	Case Control Study	1st degree	4	3.22	1.17	0.78	0.70-14.69
		Kuo (27)	2015a	Taiwan	Registry-based Cohort Study	1st degree	117	5.87	1.77	0.093	4.89-7.05
		Kuo (48)	2015b	Taiwan	Registry-based Cohort Study	1st degree	109	6.25	1.83	0.099	5.15-7.58
		Priori (33)	2003	Italy	Case Control Study	1st degree	0	–	–	–	–
		Thomsen (29)	2020a	Sweden	Registry-based Cohort Study	1st degree	61	3.56	1.27	0.13	2.76-4.58
SSc	SjD	Kuo (27)	2015a	Taiwan	Registry-based Cohort Study	1st degree	3	2.39	0.87	0.58	0.77-7.41
		Kuo (49)	2016	Taiwan	Registry-Based Case-Control Study	1st degree	4	2.77	1.02	0.50	1.04-7.35
		Alexandra (30)	2019	Israel	Case Control Study	1st degree	1	-	–	–	–
		Thomsen (29)	2020a	Sweden	Registry-Based Case-Control Study	1st degree	14	2.42	0.89	0.27	1.42-4.14
IIM	SjD	Ginn (43)	1998	USA	Case Control Study	1st degree	1	0.21	-1.55	1.48	0.011-3.88
		Kuo (27)	2015a	Taiwan	Registry-based Cohort Study	1st degree	1	0.71	-0.34	1.00	0.10-5.07
		Thomsen (29)	2020a	Sweden	Registry-based Cohort Study	1st degree	3	0.46	-0.077	0.65	0.13-1.65
MS	SjD	Nielsen (32)	2008	Denmark	Registry-based Cohort Study	1st degree	5	1.70	0.46	0.53	0.70-4.20
		Kuo (27)	2015a	Taiwan	Registry-based Cohort Study	1st degree	4	3.38	1.22	0.50	1.26-9.05
		Mescheriakova (31)	2019	Netherlands	Multigenerational Cohort Study	1st degree	1	3.29	1.19	1.48	0.18-60.21
T1D	SjD	Kuo (27)	2015a	Taiwan	Registry-based Cohort Study	1st degree	23	1.97	0.68	0.22	1.29-3.02
		Kuo (28)	2018	Taiwan	Registry-based Cohort Study	1st degree	30	1.66	0.51	0.16	1.21-2.26
GD	SjD	Thomsen (29)	2020b	Sweden	Registry-based Cohort Study	1st degree	141	1.42	0.35	0.086	1.20-1.68
HT	SjD	Thomsen (51)	2020b	Sweden	Registry-based Cohort Study	1st degree	114	1.42	0.35	0.093	1.18-1.70

FDR, first-degree relative; Year, year of publication.

SjD, Sjögren’s disease; AD, autoimmune disease; SLE, systemic lupus erythematosus; RA, rheumatoid arthritis; SSc, systemic sclerosis.

IIM, idiopathic inflammatory myopathies; MS, multiple sclerosis; T1D, type 1 diabetes; GD, Graves’ Disease; HT, Hashimoto’s Thyroiditis; Case, each AD positive with SjD FDR.

### 3.4 Meta-analysis for SjD probands with SjD FDRs

**Figure 2:**
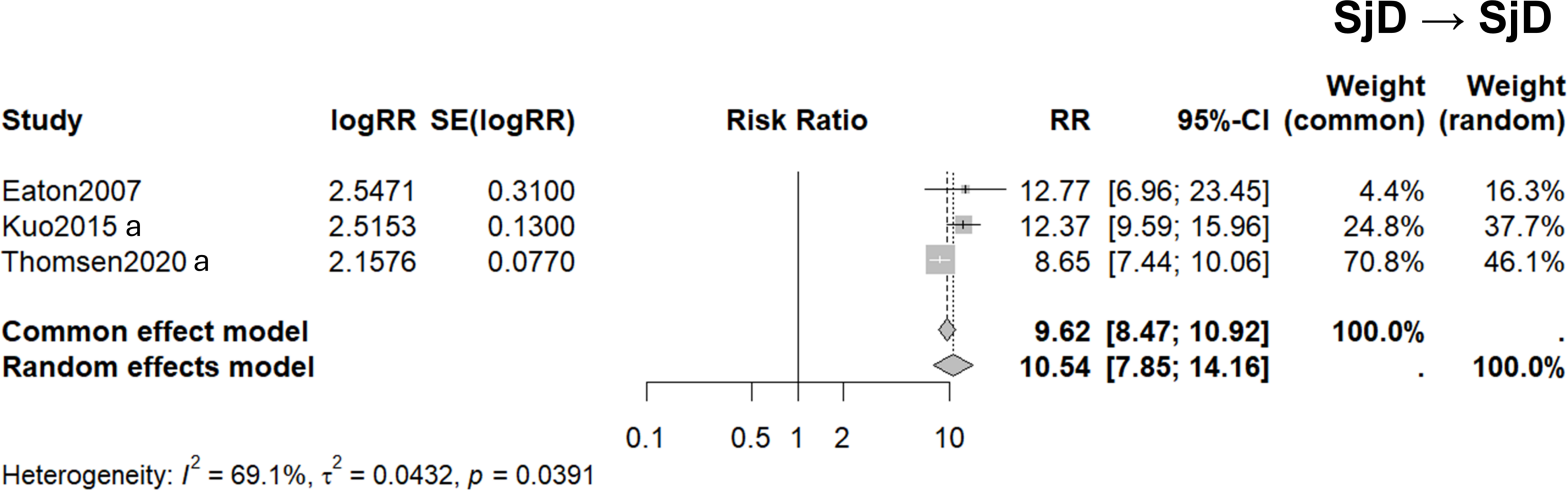
Familial risk of Sjögren’s disease (SjD) among first-degree relatives (FDRs) of probands with SjD. Arrow direction indicates disease status in probands → outcome in first-degree relatives. Three studies are listed in which both the proband and their FDRs have SjD. Forest plots demonstrate 95% confidence intervals for the relative risk ratios for each study. Pooled risks using the common and random effects models are shown. *I^2^* and *t^2^* values demonstrate study heterogeneity.

Three studies (Priori et al., (47); Kuo et al., (27); Thomsen et al., (29)) focused specifically on SjD in both probands and FDRs (27, 47, 52) (These studies represented national cohorts in Denmark (44), Sweden (29) and Taiwan (27). The heterogeneity between the studies was substantial, with an I^2^ value of 69.1%. This high level of heterogeneity suggests a considerable variability in the study populations or methodologies. A random-effects model was applied to account for this variability, resulting in a pooled RR of 10.54 with a 95% CI ranging from 7.85 to 14.16 (**Figure 2**).

### 3.5 Meta-analysis for SjD probands with discordant autoimmune diseases in FDRs

**Figure 3:**
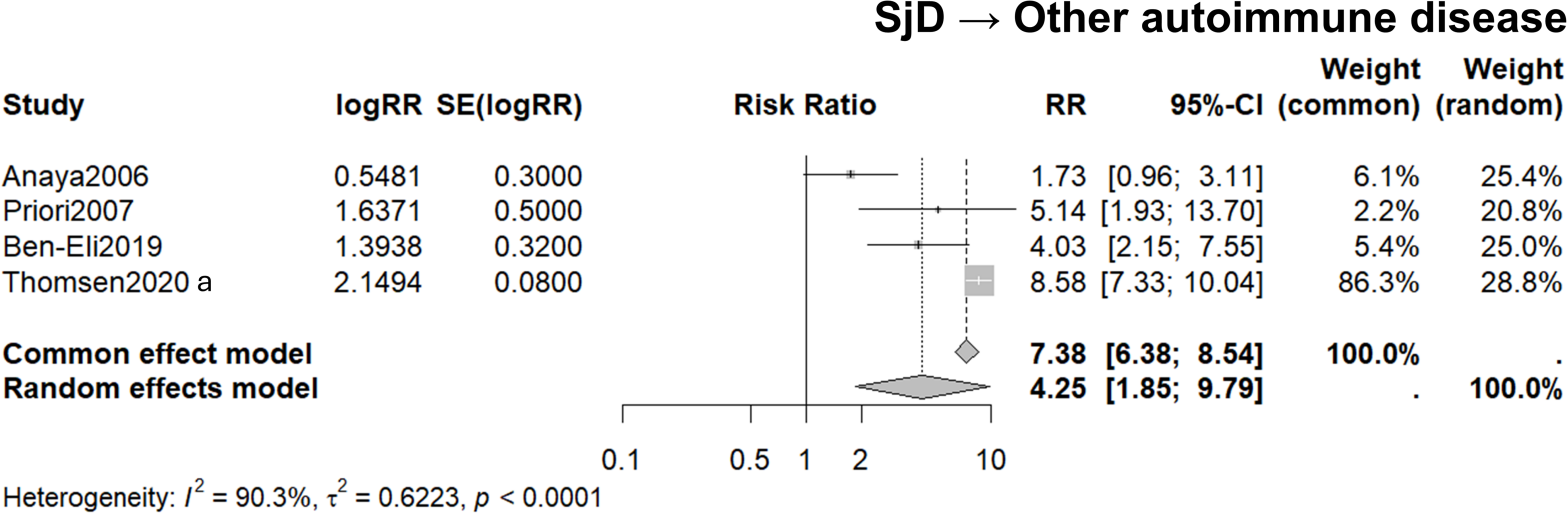
The familial risk of different autoimmune disease among FDRs of probands with SjD. List of four studies in which the proband is for SjD and the FDRs is another discordant autoimmune disease. Forest plots demonstrate 95% confidence intervals for the relative risk ratios for each study. Pooled risks using the common and random effects models are shown. *I^2^* and *t^2^* values demonstrate study heterogeneity.

Four studies (Anaya et al., (45); Priori et al., (47); Ben-Eli et al., (50); Thomsen et al., (29)) for meta-analysis were focused on SjD probands and included data for FDRs diagnosed with any other autoimmune disease. These articles included three case-control studies conducted in Colombia, Italy and Israel and one cohort study conducted in Sweden. The heterogeneity between the studies was high, with an I^2^ value of 90.3%. Meta-analysis applying a random-effects model, calculated the RR to be 4.25, with a 95% CI ranging from 1.85 to 9.79 (**Figure 3**).

### 3.6 Meta-analysis for autoimmune disease probands with SjD FDRs

**Figure 4:**
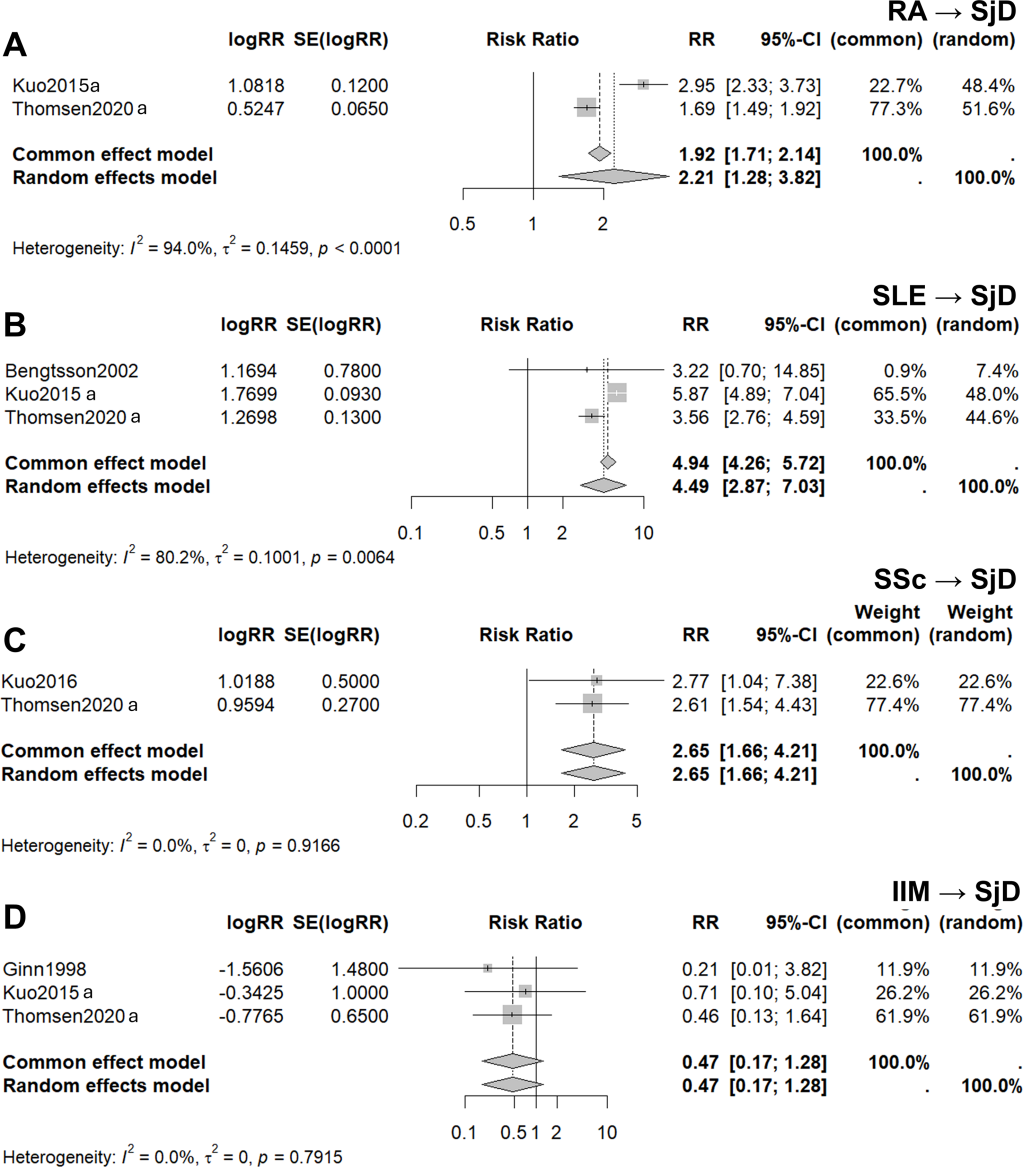
Familial risk of SjD among FDRs with other autoimmune diseases. Familial risk of SjD among FDRs of probands with **(A)** rheumatoid arthritis (RA), **(B)** systemic lupus erythematosus (SLE), **(C)** systemic sclerosis (SSc), and **(D)** idiopathic inflammatory myopathies (IIM). For each association, forest plots demonstrate the relative risk of SjD in FDRs of proband groups with the indicated autoimmune disease. Pooled risks using the common and random effects models are shown, along with *I^2^* and *t^2^* values indicating heterogeneity.

The RRs of having SjD in FDRs when a probands is diagnosed RA, SLE, SSc and IIM are analyzed in **Figure 4**. The forest plot in **Figure 4A** present compares the RR of SjD in FDRs when the proband is diagnosed with RA. This analysis was derived from two studies (Kuo et al., (49); Thomsen et al., (29)) with a large degree of heterogeneity, with an I^2^ value of 94.0%. Applying a random-effects model, the pooled RR was 2.21, with a 95% CI ranging from 1.28 to 3.82. The forest plot in **Figure 4B** presents a similar analysis, comparing the RR of SjD in FDRs when the proband is diagnosed with SLE. This analysis was derived from three studies The heterogeneity across these studies was high, with an I^2^ value of 80.2% and the random-effects model yielded an RR of 4.49, with a 95% CI ranging from 2.87 to 7.03. **Figure 4C** displays results from two studies with probands with SSc There was no heterogeneity among the studies (I^2^ = 0.00%), so both the random-effects and common-effects model was applied to produce a pooled RR of 2.65, with a 95% CI ranging from 1.66 to 4.21. Last, **Figure 4D** presents the RRs for SjD in FDRs of probands with IIM. This analysis was derived from three studies (Ginn et al., (43); Kuo et al., (27); Thomsen et al., (29)) with no heterogeneity (I^2^ = 0.00%) and both the common-effects and random-effects model produced a pooled RR of 0.47, with a 95% CI ranging from 0.17 to 1.28. Overall, the RR of SjD in relatives of individuals with SLE was the highest, followed by SSc, RA and IIM.

### 3.7 Polygenic genetic correlation of SjD with other autoimmune diseases using public GWAS summary statistics

**Figure 5:**
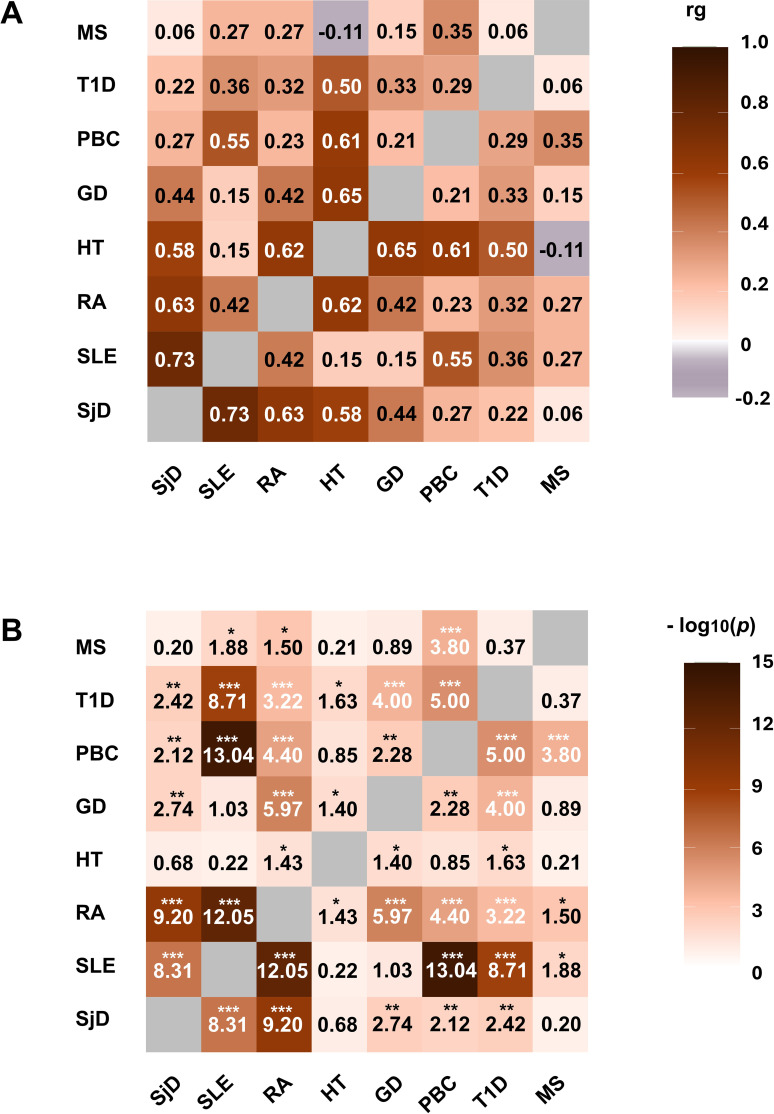
Genome-wide genetic correlations among autoimmune diseases estimated by LD score regression. Genome-wide genetic correlations (rg) among SjD, GD, HT, SLE, PBC, T1D, RA, and MS, estimated using LD score regression (LDSC) which quantifies shared polygenic effects between traits. Genetic correlations were calculated using genome-wide variants after exclusion of the HLA region (chromosome 6: 25–34 Mb). **(A)** Heatmap showing pairwise genetic correlation estimates (rg). Colors indicate the magnitude of rg, with darker colors representing stronger positive genetic correlation. **(B)** Heatmap showing the statistical significance of the corresponding rg estimates, expressed as −log_10_(P), with darker colors indicating stronger statistical support. Asterisks denote significance levels (*P < 0.05, **P < 0.01, ***P < 0.001). Diagonal cells represent self-comparisons (rg = 1) and are shown in grey.

To complement the findings from the familial meta-analysis and to evaluate shared genetic architecture at the genome-wide polygenic level, we estimated genetic correlations between SjD and other autoimmune diseases using publicly available GWAS summary statistics. Eight autoimmune diseases, SjD, SLE, HT, T1D, GD, RA, MS and primary biliary cholangitis (PBC) were included. These diseases were selected based on prior evidence of genetic or epidemiological association (34). They were classified as prototypical autoantibody-associated autoimmune diseases, and the availability of large-scale European ancestry GWAS datasets. Genome-wide genetic correlations were estimated using LD score regression (LDSC), which quantifies the extent of shared polygenic effects between traits while accounting for linkage disequilibrium structure across the genome. Pairwise genetic correlations and corresponding p values were calculated using full GWAS summary statistics for each disease pair. As shown in **Figure 5A**, the estimated genetic correlations (rg) and their statistical significance (−log_10_(P)) indicate that several autoimmune disease pairs exhibit significant genome-wide polygenic sharing. Notably, SjD exhibited significant positive genetic correlations with SLE (rg: 0.73), RA (rg: 0.63), and moderate sharing with HT (rg: 0.58) and GD (rg: 0.44). The strongest correlation was observed between SjD and SLE (−log_10_P: 8), whereas correlation with MS was minimal (rg: 0.06), indicating that SjD is genetically distant from neuroinflammatory autoimmunity. Overall, these results indicate that SjD shares substantial genome-wide polygenic architecture with both systemic (SLE, RA) and organ-specific (HT, T1D) autoimmune diseases, positioning SjD as a bridging autoimmune disease connecting systemic and thyroid-related autoimmunity.

### 3.8 HLA-dependent and non-HLA-dependent locus sharing across eight autoimmune diseases

**Figure 6:**
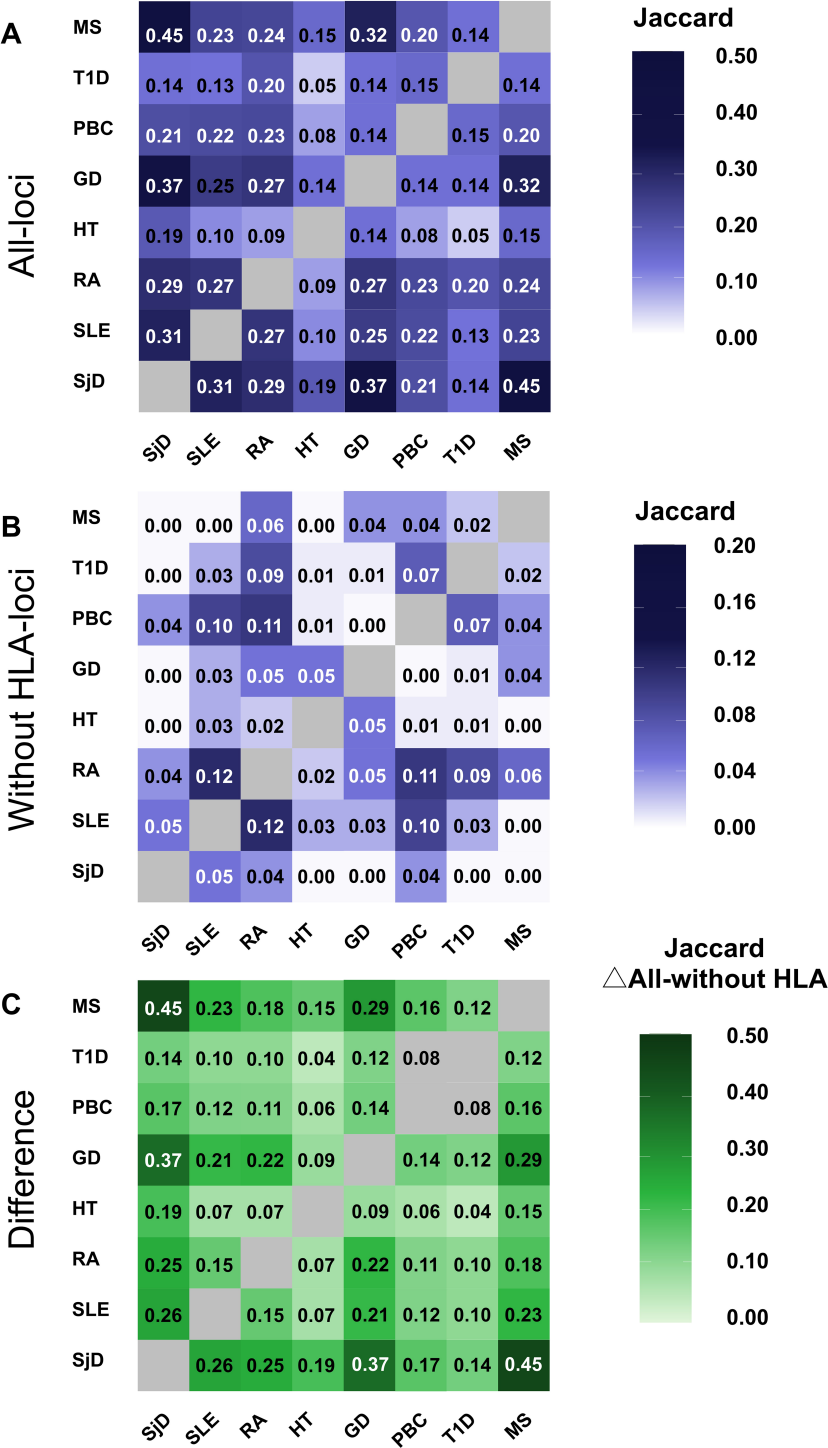
Locus-based genetic overlap among autoimmune diseases. Pairwise locus-level overlap among eight autoimmune diseases quantified using the Jaccard similarity index based on independent genome-wide significant (GWS) loci (P < 5 × 10^−8^). Independent loci were defined by merging GWS variants within ±250 kb windows. **(A)** All loci: Heatmap of Jaccard similarity calculated using all independent loci, including the HLA region. **(B)** Non-HLA loci: Jaccard similarity after exclusion of loci overlapping the HLA region (chr6:25–34 Mb, GRCh38). **(C)** Difference between all-loci and non-HLA Jaccard indices (ΔJaccard), reflecting the contribution of HLA-associated loci to locus-level sharing. Color intensity corresponds to the magnitude of locus overlap.

To quantify locus-level genetic sharing across autoimmune diseases, we next assessed overlap among significant genome-wide loci using a locus-based approach. For each disease, GWS variants (P < 5 × 10^−8^) were extracted from publicly available GWAS summary statistics and aggregated into independent loci by merging overlapping ±250 kb windows, thereby accounting for linkage disequilibrium and reducing redundancy at the variant level. Pairwise locus overlap between diseases was quantified using the Jaccard similarity index, which measures the proportion of shared loci relative to the total number of loci across disease pairs. Jaccard indices were calculated both with and without HLA loci. Using all loci (**Figure 6A**), substantial locus overlap was observed across multiple autoimmune disease pairs. SjD demonstrated prominent locus sharing with MS, and moderate sharing with GD, SLE and RA. To evaluate the contribution of the HLA region, analyses were repeated after excluding loci overlapping chr6:25–34 Mb. After HLA exclusion (**Figure 6B**), Jaccard similarity values decreased across pairs. For SjD, non-HLA Jaccard indices were markedly reduced (SjD–SLE: 0.05; SjD–RA: 0.04; SjD–HT: 0.00; SjD–GD: 0.00; SjD–MS: 0.00), indicating that most locus-level sharing involving SjD is HLA-dependent. To further delineate the relative contribution of HLA, we calculated the difference between all-loci and non-HLA Jaccard indices (**Figure 6C**). Disease pairs showing larger reductions were predominantly HLA-driven, whereas pairs retaining overlap after exclusion reflected shared non-HLA immune regulatory architecture. Importantly, genetic correlation (rg) and Jaccard similarity capture distinct aspects of shared architecture: rg reflects genome-wide polygenic covariance across all SNPs, whereas Jaccard measures overlap of genome-wide significant peaks. The discordance between these metrics indicates that strong polygenic correlation does not necessarily imply extensive sharing of discrete susceptibility loci. The reduction in Jaccard index after HLA exclusion reflects the relative contribution of classical HLA associations versus non-HLA immune regulatory regions to locus sharing across autoimmune diseases. To further explore the relationship between genome-wide polygenic correlation (rg) and locus-level overlap (Jaccard index), we compared these metrics across all disease pairs (**Supplementary Figure 1**). Using all loci, rg and Jaccard values were poorly correlated, indicating that strong HLA-driven locus sharing can inflate apparent overlap independent of distributed polygenic covariance. In contrast, after excluding HLA loci, modest alignment between rg and non-HLA Jaccard indices emerged, suggesting that non-HLA architecture more faithfully reflects shared polygenic structure. These findings highlight that genetic correlation and locus overlap capture distinct, but complementary, dimensions of shared genetic architecture.

### 3.9 Union locus analysis identifies shared and SjD-specific susceptibility regions

**Figure 7:**
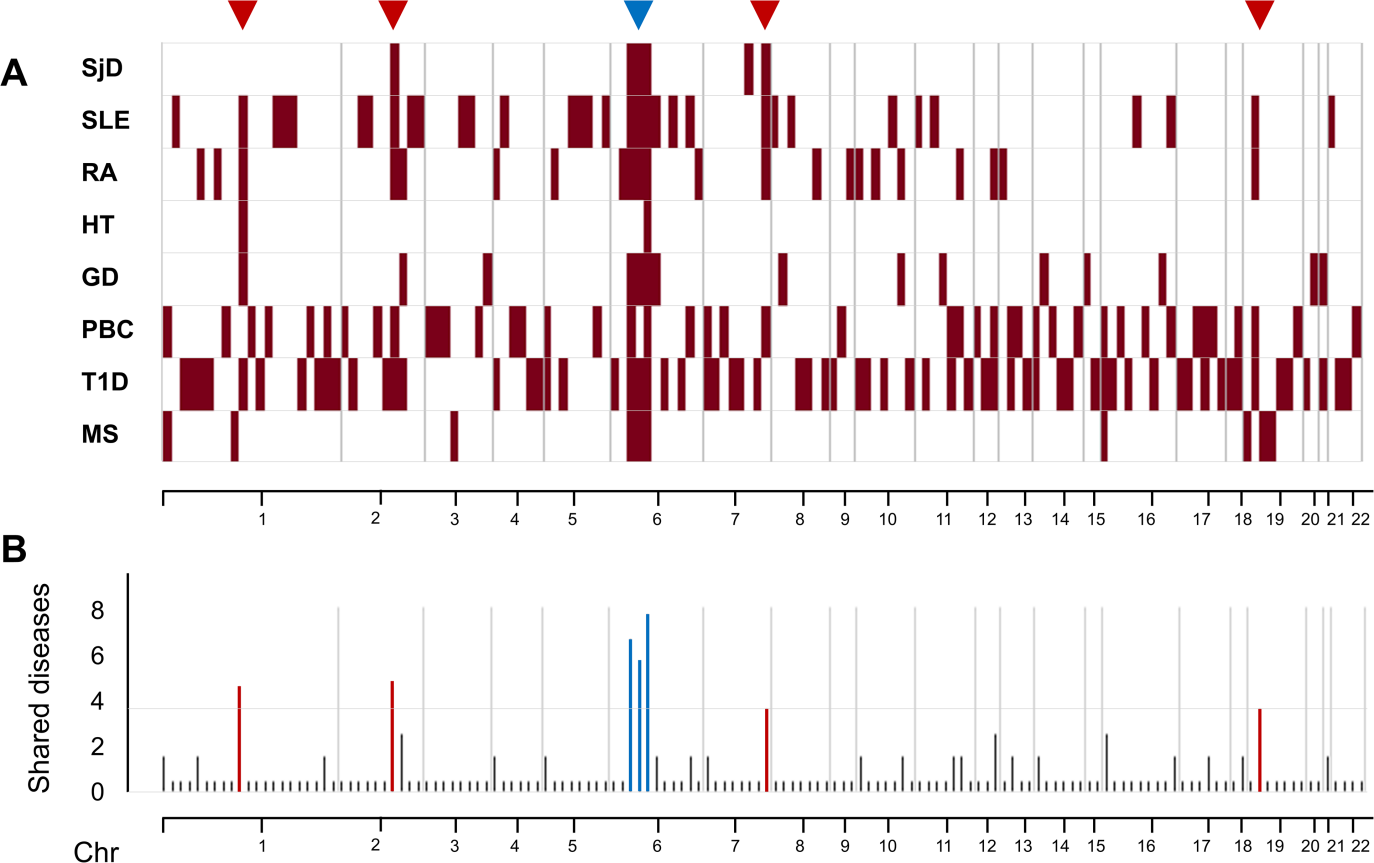
Union-based analysis of genome-wide significant loci across eight autoimmune diseases. Union-based analysis of genome-wide significant loci across eight autoimmune diseases. GWS variants (P < 5 × 10^−8^) were aggregated into independent loci using ±250 kb windows and subsequently merged across diseases to generate non-overlapping union loci. **(A)** Locus-by-disease presence matrix. Each row represents a union locus, and each column represents a disease. Red indicates the presence of at least one GWS SNP within the locus for the corresponding disease. Rows are ordered by the number of diseases sharing the locus. Inverted triangles (▽) above the plot indicate the genomic positions of representative loci highlighted in panel **(B)**. **(B)** Summary of union loci shared across diseases. Loci shared by four or more diseases are highlighted. Blue indicates loci located within the HLA region (chr6: 25–34 Mb), whereas red indicates shared loci located outside the HLA region. This visualization illustrates that multi-disease sharing is predominantly driven by the HLA locus, with limited convergence at non-HLA immune regulatory hub loci, while the majority of loci remain disease-specific.

To identify discrete genomic regions underlying cross-disease genetic overlap, we next performed a locus-level univariate analysis based on GWS variants and constructed union loci across autoimmune diseases. GWS variants (P < 5 × 10^−8^) from each disease were aggregated into independent loci by merging overlapping ±250 kb windows. Shared and disease-specific loci were summarized using a union-based framework, and overlap across SjD with GD, HT, SjD, SLE, PBC, T1D, RA, and MS(**Figure 7**). The presence or absence of association within each union locus was encoded as a binary variable for each disease and visualized as a locus-by-disease matrix (**Figure 7A**). Rows represent union loci, and columns represent diseases. This framework allows direct comparison of shared and disease-specific susceptibility regions independent of SNP-level redundancy. Consistent with the locus-based Jaccard analysis, the majority of union loci were disease-specific. Only a limited subset of loci were shared across multiple autoimmune diseases. Notably, multi-disease shared loci were concentrated within a small number of immune regulatory hub regions, including STAT4 (chr2:191 Mb), IRF5/TNPO3 (chr7:128 Mb), and PTPN22 (chr1:113–114 Mb), rather than being uniformly distributed across the genome. SjD was characterized by a small number of independent loci compared with several other autoimmune diseases. Within the union framework, SjD-associated loci were shared to varying extents with HT, SLE, and RA, while remaining largely distinct from T1D, GD, and MS. These findings support a shared core plus disease-specific layer model of autoimmune genetic architecture, in which SjD occupies a central position within a network of systemic autoimmunity rather than representing a mere subtype of a single disease. Pairwise shared locus counts are summarized in **Figure 7B**, demonstrating heterogeneity in cross-disease sharing patterns across the autoimmune spectrum.

### 3.10 SNP-level pleiotropic associations identified by PLACO

**Figure 8:**
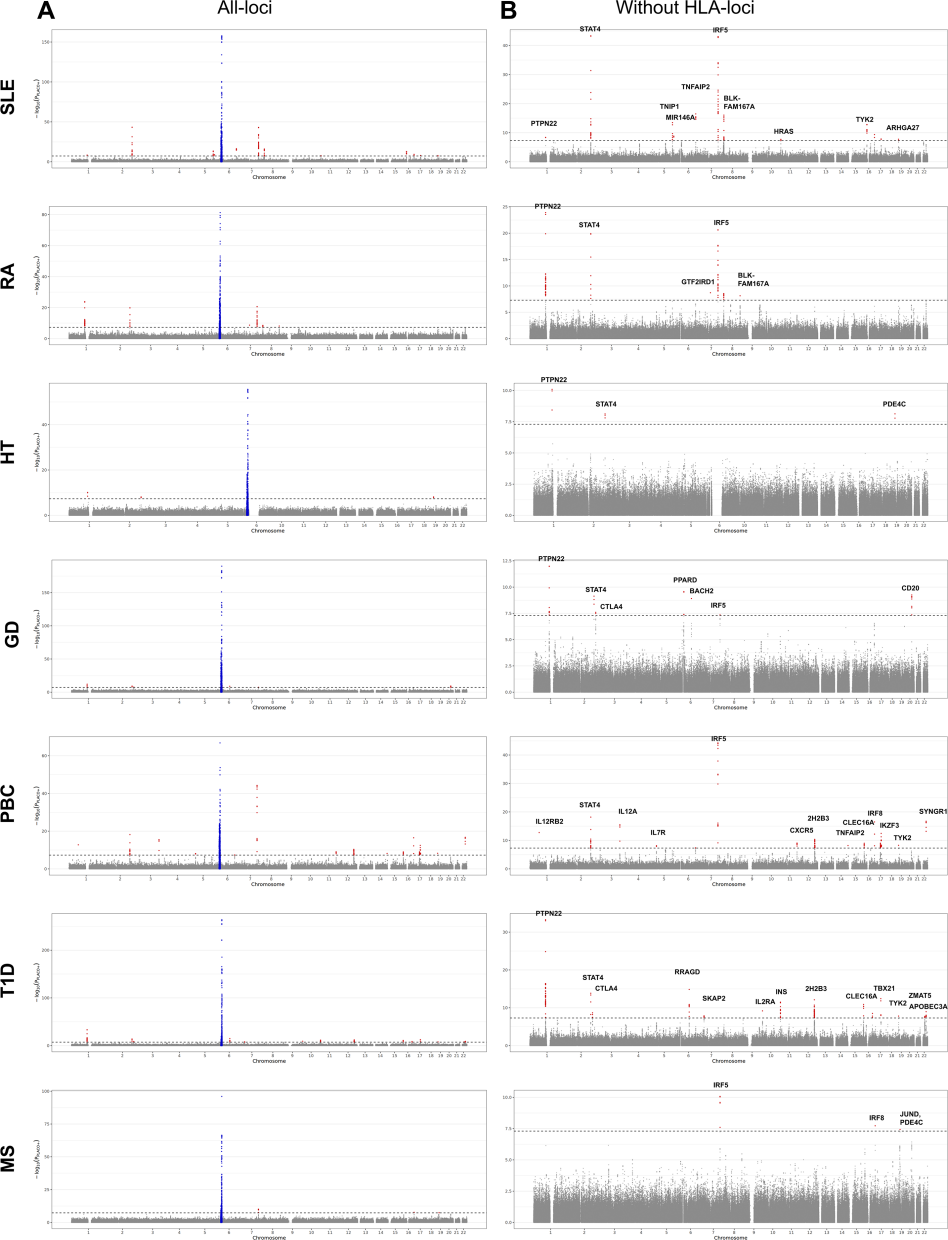
SNP-level pleiotropic signals identified by PLACO. SNP-level pleiotropy analysis between SjD and other autoimmune diseases using PLACO with cross-trait correlation adjustment. **(A)** Manhattan plot of PLACO results including all genomic regions. The y-axis represents −log_10_(P_PLACO), and the x-axis represents chromosomal position (GRCh38). The red horizontal line indicates genome-wide significance (P_PLACO < 5 × 10^−8^). Strong pleiotropic signals are predominantly observed within the HLA region. **(B)** Manhattan plot after exclusion of the HLA region (chr6:25–34 Mb). Removal of HLA substantially reduces significant pleiotropic signals, demonstrating that SNP-level pleiotropy is largely driven by the HLA region, with limited but detectable non-HLA signals.

To identify specific variants exhibiting pleiotropic effects between SjD and other autoimmune diseases, we performed SNP-level pleiotropy analysis using PLACO with cross-trait correlation adjustment. Across the seven SjD–disease pairs, genome-wide significant pleiotropic variants (P_PLACO < 5 × 10^−8^) were identified at multiple genomic locations (**Figure 8A**). The most prominent signals were concentrated within the HLA region on chromosome 6, consistent with its well-established and pleiotropic role in autoimmune susceptibility. Outside the HLA region, only a limited number of SNPs demonstrated genome-wide significant pleiotropic association, indicating that non-HLA pleiotropy is present but comparatively restricted. To quantify the extent to which pleiotropy was driven by HLA-associated signals, the analysis was repeated after excluding SNPs within chr6:25–34 Mb. As shown in **Figure 8B**, both the number and magnitude of significant pleiotropic signals were markedly reduced following HLA exclusion. Non-HLA pleiotropic signals were preferentially observed between SjD and systemic autoimmune diseases, particularly SLE, whereas overlap with MS and thyroid traits was limited. Together, these results indicate that while SjD exhibits detectable SNP-level pleiotropic sharing with other autoimmune diseases, such sharing is predominantly concentrated within the HLA region, with comparatively limited contributions from non-HLA loci. Importantly, PLACO detects shared pleiotropic signals but does not establish that identical causal variants underlie associations in both diseases; therefore, these findings are interpreted as consistent with shared genetic architecture rather than definitive evidence of shared causal mutations.

## 4 Discussion

Here, we demonstrate a high RR of familial autoimmune disease aggregation in SjD, suggesting strong genetic involvement of SjD and supporting potential shared genetic susceptibility amongst several autoimmune diseases. While previous studies have primarily focused on identifying risk factors within a single disease cohort or within families affected by SjD alone (7), our study uniquely provides a cross-disease quantitative assessment of familial aggregation between SjD and multiple autoimmune conditions. This approach allowed us to clarify not only intra-disease inheritance patterns but also the degree of shared genetic background across autoimmune diseases. This association between SjD and other autoimmune diseases has been described in numerous case reports for decades. The results of our analysis showed that the relative risk of having an FDR with SjD when the proband was also diagnosed with SjD was 10.54. This finding implies a high degree of familial clustering. The RR for SjD in FDRs was also found to be relatively high for probands with discordant autoimmune disease (RR; 4.24). While the familial association of SjD shows the highest risk, these findings demonstrate that other autoimmune diseases may aggregate with SjD and suggest possible shared genetic links between these autoimmune diseases. Importantly, extension of these familial findings to genome-wide genetic correlation analyses demonstrated that SjD shares substantial polygenic architecture with both systemic autoimmune diseases (SLE, RA) and thyroid-related autoimmunity (HT, GD), positioning SjD as a bridging autoimmune disease within the broader immune-mediated network. Consistent with this observation, comparison of Jaccard indices before and after HLA exclusion suggests that the extent of locus sharing among autoimmune diseases varies in its dependence on classical HLA regions, with some disease pairs retaining substantial non-HLA overlap. Notably, while polygenic correlation between SjD and HT was significant, locus-level overlap was largely abolished after HLA exclusion, indicating that apparent sharing may be disproportionately driven by classical HLA effects rather than distributed non-HLA susceptibility loci.

Discordant autoimmune diseases in the proband were also associated with a higher RR of SjD in FDRs, with SLE having the strongest association. The relatively high RR of SjD in FDRs of individuals with SLE (pooled RR; 4.49) may be due to their related target tissues, as both SjD and SLE involve prominent epithelial–immune interactions at affected tissues, whereas RA (pooled RR; 2.21), SSc (pooled RR; 2.65) and IIM (pooled RR; 0.47) affect different tissues. The high heterogeneity in some of the analyses (e.g., I^2^ = 96.2% for SLE and 90.9% for SSc) indicates the need for further investigation into the factors contributing to this variability, including differences in genetic susceptibility, environmental exposures, and study methodologies. PBC is frequently associated with sSjD at the individual level. In a case–control study, SjD was commonly observed among PBC patients themselves; however, SjD was not observed among first-degree relatives of PBC probands (53), despite an increased prevalence of other autoimmune conditions. This pattern contrasts with the robust familial aggregation observed between SjD and diseases such as SLE and RA in the present systematic review. Together, these findings suggest that the PBC-SjD relationship is driven predominantly by individual-level disease clustering rather than shared familial susceptibility, supporting our decision to exclude PBC from the familial aggregation meta-analysis. Population-based co-occurrence studies at the individual level have reported strong associations between SjD and several additional autoimmune diseases across diverse organ systems (9). These epidemiologic co-occurrence patterns are complementary to, but distinct from, the familial aggregation estimates synthesized here, which specifically quantify cross-disease clustering within first-degree relatives and are constrained by the availability and reporting of family-based data (16).

Prior GWAS studies comparing SjD patients with healthy controls have identified several susceptibility loci (9, 54). Notably, the HLA class II region, particularly HLA-DRB1, has been strongly implicated in SjD susceptibility (10, 11). Previous reports suggest that SjD and SLE share many common variants in the HLA region, which are more highly shared compared to RA and MS (55). The familial aggregation risk of IIM with SjD was lower compared to other autoimmune disease combinations, which may be further confounded by the clinical and pathological heterogeneity of IIM (56). To extend these familial observations to the level of shared genetic architecture, we integrated both genome-wide polygenic analyses and locus-level comparative analyses using publicly available GWAS summary statistics. GWS variants were aggregated into independent loci to account for linkage disequilibrium, enabling comparison at the locus rather than single-variant level. Consistent with prior reports, extensive locus sharing was observed within the HLA region, particularly between SjD and SLE (12, 57). However, integration of polygenic (rg), locus-level (Jaccard), and union-based analyses revealed that high genetic correlation does not necessarily translate into widespread sharing of discrete susceptibility loci. While HLA contributes disproportionately to apparent cross-disease overlap, non-HLA sharing was concentrated within a limited number of immune regulatory hub regions, including STAT4, IRF5/TNPO3, and PTPN22. These loci were repeatedly shared across multiple autoimmune diseases, suggesting convergence upon core immune pathways rather than uniform genomic overlap (58, 59). In contrast, other autoimmune diseases showed overlap with SjD that was largely confined to the HLA locus, suggesting heterogeneity across autoimmune diseases in the relative contribution of non-HLA associations.

Quantitative assessment of locus distribution further revealed that, whereas most autoimmune diseases exhibited a predominance of non-HLA loci, SjD showed a comparatively lower proportion of non-HLA GWS loci (60). This relative enrichment of HLA-dependent loci in SjD is consistent with our observation that HLA exclusion markedly reduces locus-level sharing, underscoring the dominant role of classical HLA-mediated susceptibility in shaping cross-disease overlap patterns. This finding suggests that SjD may retain a relatively stronger dependence on HLA-mediated susceptibility while also sharing selected non-HLA loci with related autoimmune diseases. Notably, patterns of locus sharing broadly mirrored familial aggregation results, supporting the biological relevance of shared genetic susceptibility (16). At the same time, the majority of loci remained disease-specific, supporting a shared core plus disease-specific layer model of autoimmune genetic architecture.

Our observation that the RR of SjD among siblings is higher than that among parent-child pairs suggests the possibility that both heightened genetic susceptibility and even shared environmental factors contribute to disease development. One possible mechanism involves polygenic inheritance, where siblings are more likely to inherit the same combination of risk alleles. In addition to gene polymorphisms, epigenetic modifications have emerged as other possible contributors to SjD pathogenesis (61). Besides genetics, there may be other explanations for familial association including shared prenatal and early life exposures, microbiome influences and similar timing of immune system development (62). Moreover, it is well established that certain autoimmune diseases, such as SSc and MS exhibit a latitude gradient, where disease prevalence increases in higher-latitude regions (21, 63). In our study, however, the observed distribution of cases did not align with a latitude gradient (data not shown). Further investigations incorporating larger cohorts and environmental exposure data will be essential to clarify these influences.

One limitation of our study relates to the racial representation. While recent studies have indicated that individuals of African descent are at particularly at high risk for SjD and other autoimmune diseases (64), our meta-analysis relied on data primarily comprising Caucasian and Asian populations. Specifically, there was a notable lack of data on African groups, thereby introducing a potential bias in racial representation. Another limitation is the variation in diagnostic criteria. While the majority of studies used the American European criteria or the ICR criteria for diagnosis of SjD, some studies relied on data collected via questionnaires. However, even in these questionnaire-based studies, follow-up by rheumatologists and/or confirmation through medical records was conducted. In addition, for the meta-analysis, the degree of heterogeneity varied markedly among the disease pairs (*I^2^* = 0–94%), with higher heterogeneity observed between SjD and RA and between SjD and SLE, and no heterogeneity between SjD and SSc or between SjD and IIM. Such variability may reflect differences in sample sizes, population structure, diagnostic criteria, or environmental factors among the included studies. Therefore, the pooled estimates should be interpreted with caution. Lastly, our study focused on only autoimmune diseases, and we did not include publications discussing additional disease associations such as B-cell non-Hodgkin lymphoma in FDRs with a higher incidence among patients with SjD (65, 66).

Overall, our study is the first to use meta-analysis to comprehensively analyze the RRs of SjD in the context of other autoimmune diseases within the same family. By integrating large-scale publicly available GWAS data with complementary polygenic and locus-level analyses, our study provides a comprehensive view of shared and disease-specific genetic architecture across autoimmune diseases centered on SjD. While extensive familial aggregation and genome-wide polygenic correlations highlight broad shared susceptibility among autoimmune conditions, locus-level analyses reveal a more constrained architecture in SjD, characterized by a limited number of key susceptibility regions with a relatively stronger contribution from the HLA locus. Taken together, these findings are consistent with a hierarchical organization of autoimmune genetic architecture, in which widespread polygenic sharing—substantially influenced by HLA-associated risk—coexists with selective convergence upon a limited number of immune regulatory hub loci, particularly between SjD and systemic autoimmune diseases such as SLE.

## Data Availability

Publicly available datasets were analyzed in this study. These data can be found in the GWAS Catalog and FinnGen Release 12 databases. Accession numbers are provided in the manuscript.

## Supplementary material

Supplementary Figure 1 Relationship between genome-wide polygenic correlation and locus-level overlap. Scatter plots comparing pairwise genetic correlation (rg) estimated by LDSC and locus-level overlap quantified by the Jaccard similarity index across autoimmune disease pairs. (A) Jaccard similarity calculated using all independent genome-wide significant loci (including the HLA region). No meaningful correlation was observed between rg and locus overlap (r = −0.085), indicating that HLA-driven locus sharing inflates apparent overlap independently of genome-wide polygenic covariance. (B) Jaccard similarity calculated after exclusion of HLA loci (chr6:25–34 Mb). Removal of HLA modestly improves correspondence between rg and locus overlap (r = 0.183), suggesting that non-HLA regions more accurately reflect distributed shared polygenic architecture. Point size corresponds to the number of overlapping base pairs between disease pairs.

Supplementary Table 1 Results of publication bias of studies in meta-analysis.
