# Supplementary material for: Familial and genetic overlap between Sjögren’s disease and other autoimmune diseases
Source: Front Immunol. 2026 Mar 26;17:1740360. doi: 10.3389/fimmu.2026.1740360 (PMC13062241; doi:10.3389/fimmu.2026.1740360)
Supplement: Supplementary Table 1 — Results of publication bias of studies in meta-analysis. [file Supplementaryfile1.docx]

**The Appendix 1. Search terms for** **PubMed.**

("Sjogren's syndrome"[MeSH] OR "Autoimmune Diseases"[MeSH] OR "Rheumatoid arthritis" OR "Systemic lupus erythematosus" OR "Lupus" OR "Multiple sclerosis" OR "Type 1 diabetes" OR ” Hashimoto thyroiditis” OR “Graves Disease” OR "Multiple sclerosis" OR “Multiple Autoimmune syndrome”) AND (“family“ OR “families“ OR “family history" OR “familial risk” OR “familial risks” OR “familial aggregation“ ) AND (“Prevalence” OR “Odds ratio” OR “Relative risk” OR “Relative risks” OR "Confidence interval" OR "Percentage“ OR “standardized incidence ratios”) NOT ("Conference Abstract"[Publication Type] OR "Editorial"[Publication Type]) And (“English”[Language])
