# Supplementary material for: Familial and genetic overlap between Sjögren’s disease and other autoimmune diseases
Source: Front Immunol. 2026 Mar 26;17:1740360. doi: 10.3389/fimmu.2026.1740360 (PMC13062241; doi:10.3389/fimmu.2026.1740360)
Supplement: Supplementary file 3 [file Table1.docx]

| **Sup-Table1. Results of Publication Bias of studies in meta-analysis.** | | | | |
| --- | --- | --- | --- | --- |
|  | **Disease** | |  |  |
| **Figure** | **Probands** | **FDRs** | **Egger’s p-value** | **Begg’s p-value** |
| Figure2 | SjD | SjD | 0.3207 | 1 |
| Figure3 | SjD | AD | 0.4987 | 1 |
| Figure4B | SLE | SjD | 0.6073 | 1 |
| Figure4C | IIM | SjD | 0.7859 | 1 |
| Year: year of publication; FDRs: first-degree relative; | | | |  |
| SjD: Sjögren’s disease; AD: autoimmune disease; | | | |  |
| SLE: systemic lupus erythematosus; IIM: idiopathic inflammatory myopathies | | | | |
